# Zero-shot prediction of drug responses using biologically informed neural networks trained on phosphoproteomic timeseries

**DOI:** 10.1371/journal.pcbi.1014100

**Published:** 2026-03-18

**Authors:** Konstantinos Antonopoulos, Olof Nordenstorm, Avlant Nilsson

**Affiliations:** 1 Department of Protein Science, SciLifeLab, KTH Royal Institute of Technology, Stockholm, Sweden; 2 Department of Cell and Molecular Biology, SciLifeLab, Karolinska Institutet, Stockholm, Sweden; Clemson University, UNITED STATES OF AMERICA

## Abstract

Cellular signaling is driven by complex, dynamic phosphorylation networks that control growth and survival, and their dysregulation underlies diseases such as cancer. Although modern mass spectrometry enables large-scale quantification of phosphoproteomic responses over time, these measurements remain descriptive and cannot by themselves predict how signaling will evolve under perturbations. Here, we extend a biologically informed recurrent neural network framework (LEMBAS), to learn time-resolved phosphoproteomic trajectories. We introduce two interpretable modules; a phosphosite mapping that links signaling nodes to measured phosphorylation sites and a monotonic time mapping that aligns continuous experimental times to discrete signaling steps. Using synthetic benchmarks and an EGF-stimulation dataset with inhibitor treatments, the model accurately interpolates unseen time points and predicts drug-induced phosphoproteomic responses in a zero-shot setting, outperforming naïve and fully connected baselines. Importantly, the model identifies both canonical and non-canonical signaling effects, including modulation of the transcription factor FOXO3:S7 (from the PI3K/AKT pathway) by drugs affecting PTPN11 (from the RAS/ERK pathway). By combining mechanistic priors with deep learning, our framework provides a scalable approach to interpret and predict dynamic drug responses from phosphoproteomic data.

## Author summary

Cells constantly adjust their behavior in response to signals from their environment, and many drugs work by altering these communication networks. Measuring these changes directly is expensive and time-consuming, so we set out to build a computer model that can make accurate predictions without needing new experiments for each drug. We trained a neural network using large datasets that track protein modifications over time after cells are stimulated. The model uses prior biological knowledge about how proteins interact, allowing it to connect molecular events to drug effects. Remarkably, it can make “zero-shot” predictions; that is, it can predict the effect of drugs it has never seen before. We show that the model can capture both expected and surprising drug responses, and it can even suggest new links between signaling proteins. Our approach demonstrates how combining biological knowledge with modern machine learning can improve the prediction of cellular responses and may ultimately accelerate drug discovery and personalized medicine.

## Introduction

Cancer is a disease of dysregulated cellular signaling. Healthy cells rely on tightly controlled signaling networks to govern proliferation, apoptosis and differentiation [1]. Cancer repurposes normal signaling pathways, enabling cells to evade growth control and resist cell death [2,3]; recurrently implicated modules include PI3K, AKT, mTOR, and RAS/MAPK, which can form self-sustaining loops that drive progression [4,5]. Because these pathways operate largely through post translational modifications such as phosphorylation, the dynamic phosphoproteome offers a direct window into how cancer cells respond to perturbations and therapy [6,7]. Recent advances in mass spectrometry now permit quantification of tens of thousands of phosphorylation events across multiple times, enabling system level measurement of signaling dynamics [8].

Yet, even with these advances, phosphoproteomic measurements alone provide only a snapshot of signaling states. They reveal which pathways are active at a given time but cannot, on their own, predict how the system would evolve under different conditions [8,9]. A promising way forward is to use such datasets not in isolation, but as training sets for dynamic cell models that capture causal interactions and predict cellular responses under unseen conditions [10,11].

Treating signaling as a dynamical system is conceptually useful but practically difficult. While Ordinary differential equation models provide detailed causal insight, their application at large scale is often constrained by parameterization, incomplete knowledge, and experimental coverage [12,13]. Logic based and Boolean models reduce parameter needs and produce qualitative insight for large networks but require extensive curation and by design cannot resolve fine grained quantitative and temporal dynamics [14,15]. This motivates complementary data-driven approaches that can integrate prior knowledge while remaining flexible. Hybrid and modular strategies that combine mechanistic structure with learning-based components represent a promising direction.

At the same time, deep learning architectures have matured to provide powerful modeling capacity. This includes recurrent neural networks (RNNs) that are a class of neural models that processes sequential data by maintaining a hidden state that is updated at each time step, thereby capturing temporal dependencies and making it suited to model time‑resolved phosphorylation dynamics. However conventional machine learning methods are often black box and do not incorporate existing knowledge of cellular wiring, which limits mechanistic interpretability and may require substantial amount of data to relearn [16,17]. Biologically informed neural networks address this gap by embedding curated signaling networks directly into model architectures, reducing the number of free parameters and improving interpretability while retaining much of the flexibility of data driven approaches [11,18].

The Large-scale knowledge-EMBedded Artificial Signaling-networks (LEMBAS) framework exemplifies this approach [19]. LEMBAS casts signaling as an interpretable RNN constrained by curated prior knowledge of the cellular signaling network from databases, e.g., OmniPath [20] and demonstrates accurate prediction of transcription factor activity from ligand stimulation. Nonetheless, LEMBAS and related methods have mostly been applied to steady state end points rather than rich time series data of phosphorylation, leaving acute drug induced dynamics under exploited. Acute signaling responses are central to drug action because they determine whether an inhibitor blocks its target or instead activates compensatory pathways that weaken its effect [21,22].

Here we extend the LEMBAS framework to model time resolved phosphoproteomic trajectories. We preserve the prior knowledge constrained recurrent core while adding two new modules: a phosphosite mapping that converts node level activity to measured site intensities, and a monotonic time mapping that aligns continuous experimental times to the discrete steps of the RNN. Both modules are designed to train end to end with the recurrent backbone and to retain biological interpretability. Using synthetic benchmarks and a public time series dataset of the phosphoproteomic response to EGF stimulation and single timepoint drug responses [23] we show the model can infer held out time points and predict drug specific differential responses in a zero-shot setting, enabling mechanistic interpretation of on target and off target signaling effects.

## Results

### Extended LEMBAS for time-resolved phosphoproteomics

**Fig 1:**
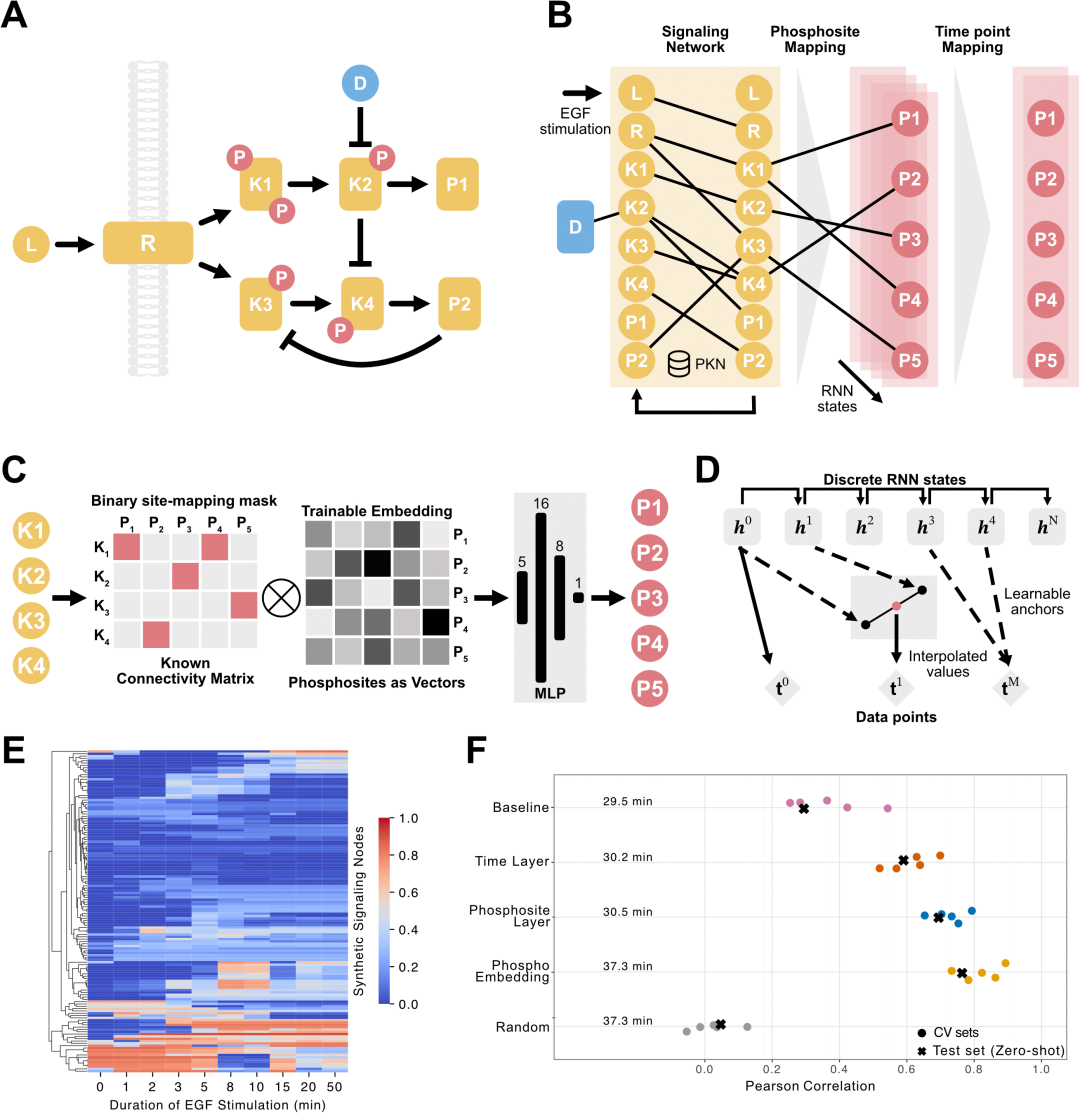
Extension of the LEMBAS framework to time-resolved phosphoproteomics. **A.** Illustration of a minimal signaling network including ligand (L), receptor (R), kinases (K1–K4), non-kinase signaling proteins (P1, P2) and kinase inhibitor (D). **B.** The model architecture mirrors this structure, ligand and drug inputs drive an RNN whose signal propagation is constrained by the prior-knowledge network (encoded as an adjacency matrix with trainable weights); the hidden state is feed into a layer that maps signal states to phosphosites, which in turn is mapped to specific timepoints. **C.** The phosphosite mapping layer connects each signaling node only to its own phosphosites that are identity-coded with a trainable a low-dimensional embedding (five dimensions), and a multilayer perceptron is used to (non-linearly) transform the signal back to a single phosphosite output. **D.** The time-point mapping layer: a set of learnable anchors is constrained to be positive, monotonic and with the first fixed at the first RNN step. Soft indexing of the two nearest integer RNN steps interpolates the value at each non-integer anchor. **E.** Generated phosphosite intensities across time points for EGF-stimulation data, only displaying variable sites (with standard deviation > 0.001). **F.** Ablation results across model configurations. Circles are the five cross-validation folds; X’s correspond to zero-shot evaluation. Average training times are also displayed.

We first extended the LEMBAS framework to handle time resolved phosphoproteomics data (see Methods), as conceptually illustrated with a minimal signaling network (Fig 1A). We encoded the network structure (from OminPath) in a weight matrix for molecular interactions and linked each drug to its protein target via prior knowledge (Fig 1B). The model could then be trained on time-resolved phosphoproteomic responses to ligand stimulation, where the inputs are experimental conditions (stimulation and presence of ligands or drugs) and the outputs are changes in phosphosite-level abundance over time. During training, both the network and experimentally observed phosphosite trajectories were provided. After training, the model could be queried by specifying a perturbation (e.g., drug target inhibition) without providing phosphoproteomic measurements to output predicted phosphosite responses consistent with the learned signaling dynamics. The model was adjusted to return the full sequence of recurrent neural network states rather than only the steady state representation used in the original LEMBAS. We used a GPU optimized reimplementation that replaces sparse CPU operations with dense GPU friendly operations and that runs full backpropagation through time. This reimplementation is described in an independent manuscript [24].

We map signaling node outputs to measured phosphosites via a masked layer with a unique trainable embedding per site (Fig 1C). Specifically, a binary selection matrix enforces the node-site correspondence and is multiplied with a low dimensional embedding (five dimensions). The resulting vectors are passed through a multilayer perceptron that collapses each embedding into a scalar phosphosite intensity. This design lets the model learn (potentially) nonlinear relations between the activity of each signaling node and its corresponding phosphosites, while enforcing that the phosphosite level prediction only depends on the state of the corresponding signaling node. Importantly, this relies on phosphosite-protein assignments to be provided in the dataset. We validated the phosphosite mapping in isolation (unit tests reported in S1 Fig). Results show that the embedding together with at least one hidden layer can recover a wide range of possible relations between signaling nodes and phosphosites. This embedding framework provides the flexibility to model complex node-to-site relationships, using the data-driven mask to anchor these connections to the underlying signaling architecture (S2 Fig).

To align RNN steps with experimental sampling we added a monotonic, differentiable time mapping layer (Fig 1D). We assign one trainable anchor per time point, constrain anchors to be positive and strictly increasing, and fix the first anchor to the first RNN step. After scaling, each anchor becomes a non-integer step; for example, an anchor of 0.82 for the 1-minute point is realized by fetching the RNN outputs at steps 0 and 1 and interpolating between them to compute the value at 0.82. Unit tests of the time mapping module are reported in S3 Fig, and the behavior of the mapping under different α (scaling) and u_raw (normalization) values is shown in S4 Fig. In summary the time mapping handles a broad set of realistic temporal patterns and is robust except when the upstream module produces a flat output time series, in which case a unique mapping does not exist, and timing information cannot be recovered.

### Synthetic benchmark & ablation study

To adapt LEMBAS to time resolved phosphoproteomics we first generated synthetic datasets that mirror the structure of experimental data (Fig 1E). The synthetic data generation pipeline used to develop and benchmark the model is summarized schematically in S5 Fig. We then performed a systematic ablation study under a five-fold cross-validation scheme and in a zero-shot setting to isolate the impact of each new module (Fig 1F). Hyperparameter choices for the GPU-optimized LEMBAS implementation were guided by our prior systematic analysis reported in Nordenström et al. [24]. In particular, we adopted those recommended settings for key structural regularizers, including L2 weight regularization and spectral radius constraints. For optimization-related parameters, such as learning rate and number of training epochs, we selected values that consistently produced smooth loss decay and stable convergence without oscillations.

Starting from a baseline model with the same structure as the model in the original LEMBAS publication, we sampled 10 equally spaced time points from the RNN steps and implemented all signaling-protein-to-phosphosite mappings as fixed identity weights. This core recurrent architecture provided a baseline level of performance. Adding the monotonic time-mapping layer improved accuracy and robustness, while introducing a site-specific scalar mapping produced further gains. The masked embedding layer achieved the highest performance, showing that each successive module captures additional variance in the data compared to simpler alternatives. As a control, we trained the full model on data with randomly shuffled sample labels and observed negligible performance (r_CV_ = 0.02 ± 0.07, r_test_ = 0.04), confirming that the model captures genuine signal rather than artefacts.

To assess the temporal sensitivity of the model under controlled conditions, we performed a down-sampling analysis on synthetic data, focusing on the first 30 RNN steps where the primary signaling dynamics occur. We measured interpolation performance as a function of the percentage of uniformly spaced timepoints provided during training (S6 Fig). Even with a small number of timepoints (six anchors), the model substantially outperformed the edge case of training on only the first and last timepoint. With twelve timepoints, performance exceeded r = 0.8, and accuracy increased steadily with additional anchors, approaching r ≈ 0.95 when the majority of timepoints were included, corresponding to near-complete reconstruction of the training trajectories.

To investigate how the number of drug perturbations in the training set influences the model’s predictive power, we performed a scaling analysis using the synthetic data (S7 Fig). We employed the same leave-one-out cross-validation strategy where, for a given training size, we iteratively trained the model on a subset of drugs and evaluated its performance on a held-out, “zero-shot” drug. While we found that data from 5 drugs was sufficient to make predictions with marked zero-shot performance (r ≈ 0.55), we observed that performance improves as the variety of drugs in the training set increases to 15 (r ≈ 0.75), while after 100 the mean correlation plateaus at approximately r = 0.80. We reason that the strong performance also in the low sample setting is likely due to phosphoproteomic data being a near direct observation of the signaling state, thereby providing detailed information about the local input-output relationships, which has to be learned implicitly in the transcription centric setting for which LEMBAS was originally developed.

### Time-point inference from phosphoproteomics data

We then transitioned to train a model in this configuration on real world data. For this we used time-resolved data from EGF-stimulation experiments on non-transformed MCF10A cells [23]. The training loss decreased smoothly and plateaued by ∼2250 epochs (the full loss curve is provided in S8 Fig). To ensure that this model focused on responsive signaling, we pruned the OmniPath network to include only nodes that where reachable from the EGF receptor. Without this pruning, nodes that are unconnected to the stimulation input would, by construction, remain at a constant activity level, and fail to capture any observed variance. To test if this restriction was well founded, we examined the distribution of experimental variance and found that phosphosites with links to the EGF receptor (EGFR) in OmniPath indeed exhibited higher variance than unconnected (0.4710 vs 0.3310; Kolmogorov-Smirnov p-value: 5.1905e-04; S9 Fig). This provided validation that the model was well set up to capture the bigger effects in the data, however, the variance in non-connected nodes also suggests the presence of non-modelled effects in the data. It should also be noted that the RNN based architecture makes the model inherently capable of modelling feedback loops present in the prior knowledge, thereby allowing the majority of nodes to be retained by the pruning process.

**Fig 2:**
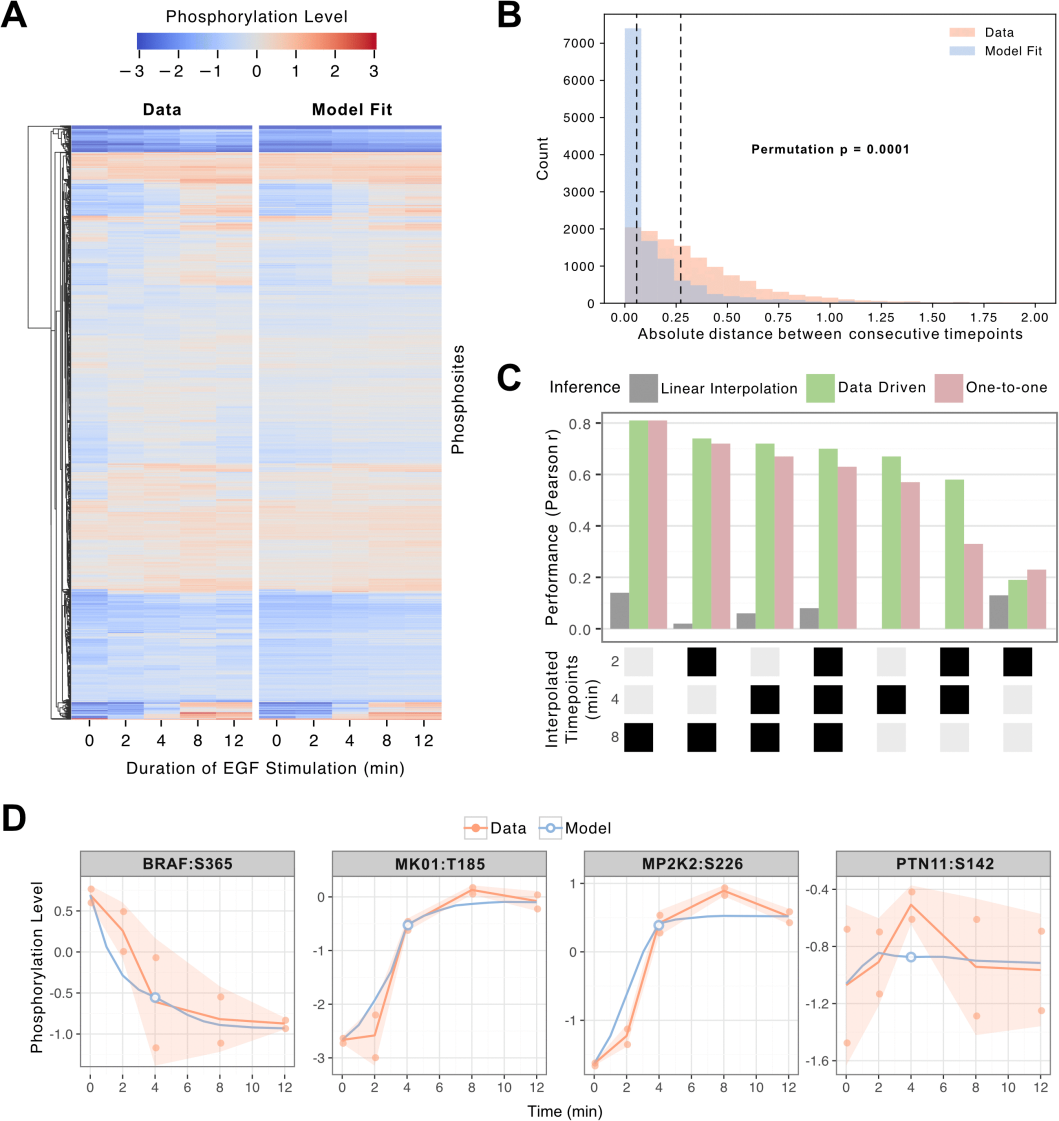
Model performance on interpolation tasks for time-resolved phosphoproteomics data. **A.** EGF-stimulation phosphoproteomics data and model fits across time points, values clipped between -3 and 3. **B.** Distribution of absolute distances between consecutive timepoints for the experimental data (median = 0.27, mean = 0.38) and model fits (median = 0.05, mean = 0.17). Dashed lines indicate the medians. The difference between both distributions is statistically significant (permutation test, 10,000 resamples, p = 0.0001). **C.** Comparison of interpolation performance on held-out time-points between linear interpolation, anchor estimation using data from a single phosphosite (GAB1:S266s), and one-to-one mapping. Black boxes on the X-axis indicates the time points held out during training. **D.** Selected examples of time series data compared to model fits and predictions at the 4 min time point. Lines indicate means; ribbons standard deviations.

The trained model accurately fits observed trajectories while exhibiting a slightly conservative behavior, meaning that fitted changes tend to be smaller than their respective measurements (Fig 2A). To quantify this conservative effect, we inspect the distribution of absolute distances between consecutive timepoints for both data and model fits, showing that the model’s temporal increments are narrower and more restrained than the experimental measurements (Fig 2B). This is an established behavior in deep learning models where they frequently track the mean of the replicates when following a trend [25,26]. In this context, it reflects a tradeoff between robustness and sensitivity: the combination of the monotonic time mapping, embedding based phosphosite mapping, and regularized training objective promotes smooth, robust estimates and reduces overfitting, but at the cost of suppressing extreme responses.

To test whether the model captures reasonable trends between measured timepoints rather than fitting only to observed anchors, we evaluated its ability to interpolate intermediate timepoints. To assess this, we held out different combinations of time points during training and then inferred them post hoc (Fig 1C). We compared three approaches: linear interpolation; a data-driven method that estimates the time points for the anchors from time series data from a single phosphosite (GAB1:S266) to infer the remainder; and a one-to-one mapping of RNN steps to time points. Linear interpolation failed in almost all cases, while the model-based methods agree closely, with the data-driven approach typically outperforming one-to-one mapping. The best single-point prediction occurred at 8 min (r = 0.81), while the one-to-one method at 2 min was fairly poor (r = 0.23). Full interpolation (training only on 0 min and 12 min) achieved r = 0.70 across all datapoints, the results for all individual combinations are provided in S10 Fig. It has been noted that the comparably low performance at the 2 min time point, can be explained by a delayed activation of downstream ERK and RSK signaling following EGF stimulation, for which many substrates are not yet robustly regulated at 2 min [23], thereby limiting the amount of coherent signal available for learning. We find that the variance at 2 min was less than half of that observed at 8 min (0.2307 vs 0.5111; S11 Fig), indicating substantially weaker signal amplitude at the earlier timepoint. Because benchmarking is performed on baseline-subtracted trajectories, this low dynamic range amplifies the impact of measurement noise, leading to weak correlations even when predictions are qualitatively reasonable. In contrast, later timepoints exhibit stronger regulation, resulting in more stable and informative correlation estimates.

We finally inspect the time series data for the two experimental replicates and model output when interpolating the 4 min time point. In three cases, the model closely follows the mean of the two replicates. As a control feature, we selected PTPN11:S142 that seemingly exhibited no true signal. For this feature the model correctly returns an almost flat profile, consistent with the measurements across five timepoints, which showed no temporal trend between replicates (linear mixed-effects model, p = 0.939), indicating that the variation reflects baseline noise rather than meaningful dynamics.

### Zero-shot prediction of drug-specific phosphoproteomics responses

We next evaluated the best configuration for predicting the effects of drugs in a zero-shot setting. The model was trained on EGF time-series data and DMSO static data at 12 min, along with static 12 min data for three out of four inhibitors. Each inhibitor was linked to its protein target via prior knowledge; SHP099 → PTPN11 (SHP2), LY294002 → PIK3CA, PIK3CB, PIK3 CD, U0126 → MAP2K1, MAP2K2, and SL0101 → RPS6KA1. The model was then used to predict the left-out inhibitor, relying solely on the trained signaling representation and prior-knowledge connectivity.

**Fig 3:**
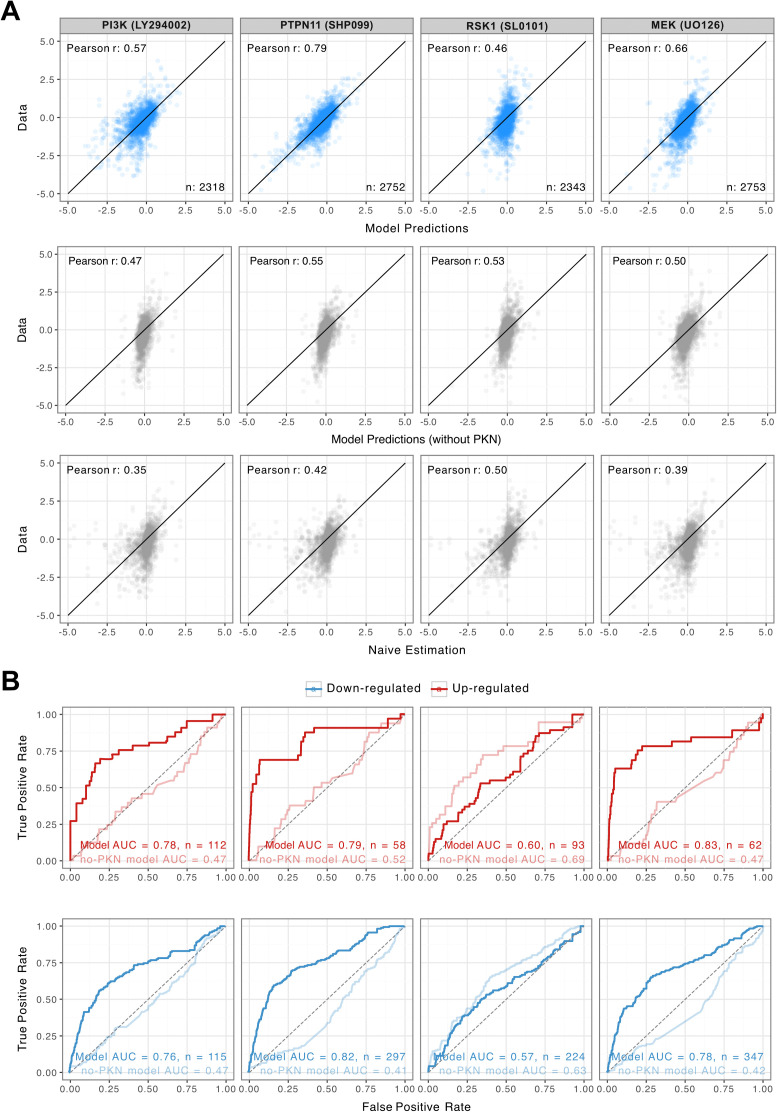
Zero-shot prediction of drug-specific phosphoproteomics responses. **A.** Model predictions versus experimental differential phosphorylation in a zero-shot setting after EGF control subtraction. Top row: biologically informed model using the OmniPath prior-knowledge network; middle row: model without prior-knowledge network (fully connected signaling layer); bottom row: naïve baseline estimated as the mean response of the remaining drugs. **B.** ROC curves for identifying up- and downregulated phosphosites using the biologically informed model (dark lines) and the model without prior knowledge (light lines). AUC values are reported for each drug.

To avoid including the basal effect of EGF stimulation in the evaluation, the EGF control data and corresponding model fits at 12 min were subtracted from the measurements and predictions respectively before calculating the Pearson correlation, i.e., measuring the correlation in differential phosphorylation (Fig 3A). In general, the performance was in line with the performance on synthetic data for a similar sample size. We also provide the raw uncorrected predictions in S12 Fig; however, these predictions don’t account for differences in basal phosphorylation state and were thus strongly inflated (r > 0.85 for all drugs). It should be noted that by restricting the model the signaling cone downstream of EGF, we have ensured that the model is evaluated only on the components that it is mechanistically capable of affecting. We tested the influence of this and find that the experimental data for these sites still fluctuate, presumably due to processes not captured by the model, thereby appearing in as a line of points at a predicted value of zero in unpruned simulations (S13 Fig).

To assess robustness and quantify model uncertainty, we repeated the zero-shot evaluation using ten independently trained models with different random initializations. Predictions were highly stable across runs as the distribution of coefficients of variation followed a decreasing exponential profile, with approximately 80% of phosphosite-sample combinations exhibiting less than 13% variation (S14 Fig). Importantly, the resulting Pearson correlations varied by less than ±0.01 across runs, indicating that the reported performance is not sensitive to random initialization (S15 Fig).

We established two basic baselines to account for unspecific effects of the drugs. A naïve baseline calculated as the mean of the other three inhibitors. For three of the four drugs, our model significantly outperformed the baseline (bootstrap p < 10 ⁻ ⁵), demonstrating the benefit of the prior-knowledge network. To further benchmark the contribution of the prior-knowledge network, we repeated the zero-shot evaluation using an otherwise identical model in which the signaling network was fully connected and thus no prior-knowledge adjacency matrix was applied (a model without PKN). While this model captured some global trends and performed better than the naïve baseline, performance was consistently lower than the biologically informed model across the same 3 drugs (Fig 3A).

The highest accuracy was obtained for the PTPN11 inhibitor (R = 0.79), this is likely due to its strong mechanistic overlap with the MEK inhibitor (pairwise correlation = 0.90) and their close proximity in the OmniPath-derived network, enabling efficient transfer of information. In contrast, the MEK inhibitor predictions were less accurate despite this similarity, suggesting that the network architecture and data distribution may favor certain propagation patterns. In this case, the PTPN11 position within the RAS–ERK pathway (upstream of RAS) may allow more effective indirect inference from the MEK inhibitor than vice versa. Another contributing factor may be the nature of their targets: the MEK inhibitor targets both MEK1 and MEK2, which are broad signaling hubs, whereas PTPN11 inhibitor specifically targets PTPN11, potentially making the signal propagation more distinct and easier for the model to capture.

The RSK1 inhibitor, showed no improvement over the baseline nor the model without PKN. This inhibitor has very low or negative correlations with all other drugs (pairwise correlations: -0.05, -0.05, and 0.01) and we also speculate that it lies in a sparsely connected region of the network, thereby limiting the ability of the prior-knowledge constraints to bridge from available training examples. Notably, the naïve baseline performed relatively well for this inhibitor despite the lower pairwise correlations with the other samples. This likely arises because the RSK1 inhibitor sample signal is less correlated with the EGF control than other drugs (R = 0.49 vs R = 0.57 ± 0.02), so control subtraction removes less shared variance. As a result, more of the true variation is preserved in the benchmarking procedure, giving the naïve baseline a higher post-subtraction correlation without reflecting mechanistic similarity.

We further assessed the model’s ability to predict up- and downregulated phosphosites for each drug. To avoid quantifying generic signaling effects that where shared by all drugs, both experimental measurements and model predictions were centered by subtracting the mean value of each phosphosite across the training samples prior to classification, and a fold-change threshold of 1 in both directions was applied to label phosphosites as either clearly up- or down-regulated. The predictions were treated as continuous scores to generate ROC curves and compute AUC values. For this test, we found that the biologically informed model outperformed the model without prior knowledge for three of the four drugs, consistent with the correlation-based benchmarking results (Fig 3B).

### Model-driven identification of non-canonical signaling

**Fig 4:**
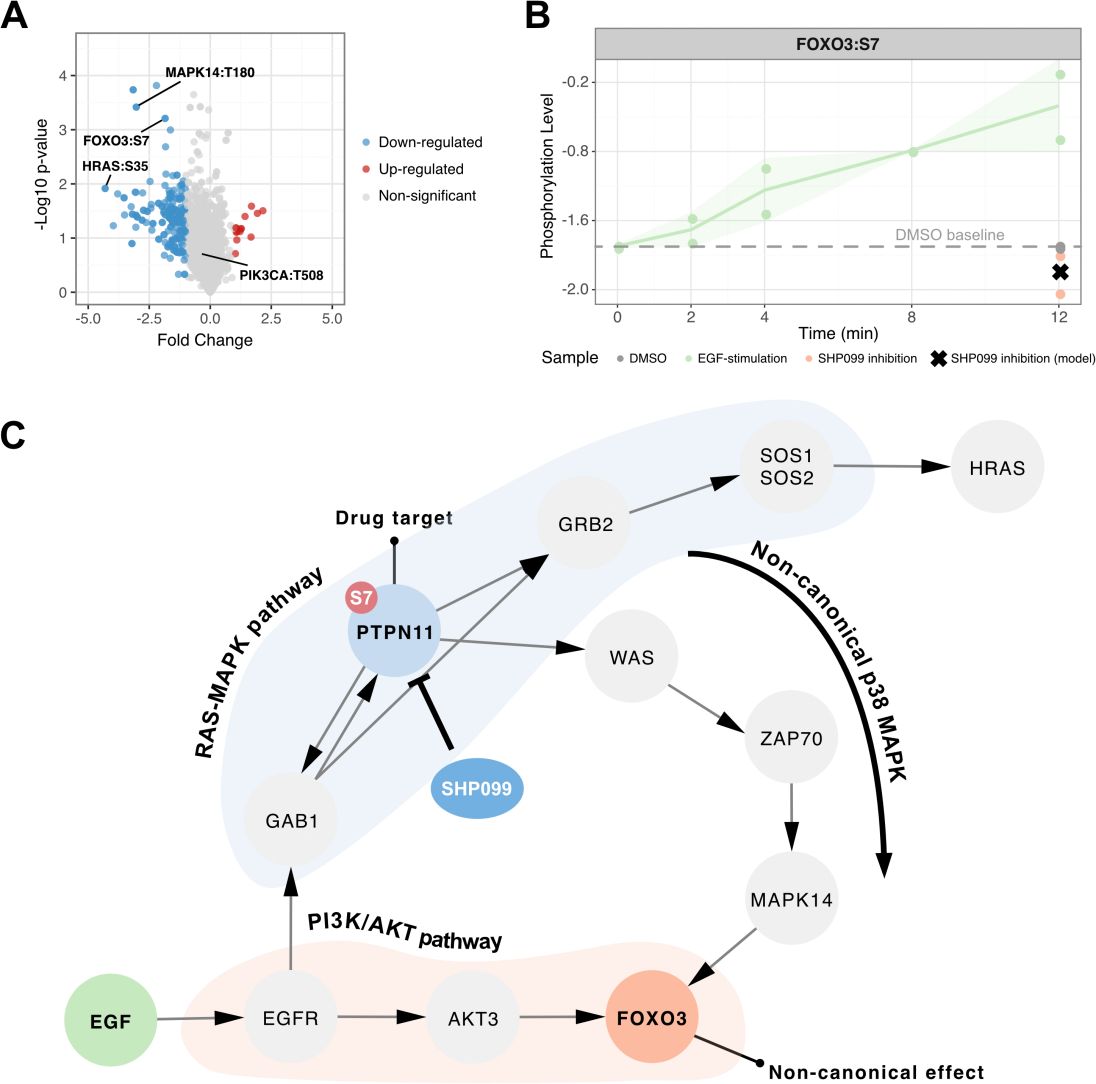
Network analysis of non-canonical signaling. **A.** Volcano plot of differentially expressed phosphosites between EGF-stimulation and SHP099 inhibition. Relevant phosphosites (FOXO3:S7, RASH:S35, MAPK14:T180, and PIK3CA:T508) are highlighted. **B.** Phosphorylation levels of FOXO3:S7 across two replicates. Full time series shown for EGF-stimulation; static data at 12 min for DMSO and SHP099 inhibition. Line indicates means; ribbon shows standard deviations; dashed line indicates DMSO-baseline; x shows the model prediction for the perturbed sample. **C.** Network visualization of the canonical RAS/MAPK pathway containing PTPN11 (SHP099 target), the PI3K/AKT pathway with FOXO3, and the potential crosstalk via p38 MAPK.

To investigate the practical utility of a model trained on phosphoproteomic data, we analyzed non-obvious predicted effects of the PTP11 inhibitor drug (SHP099), which was the one for which the model showed best performance. Specifically, we focus on its downstream effects on the phosphosite FOXO3:S7 (Fig 4A). Phosphorylation of FOXO3 at Serine 7 (FOXO3:S7) has been implicated in various cellular processes. For instance, p38-mediated phosphorylation of FOXO3:S7 is essential for its nuclear relocalization in response to doxorubicin, indicating a role in the DNA damage response [27]. Additionally, FOXO3:S7 phosphorylation is associated with mitochondrial localization, suggesting a role in mitochondrial dynamics and function [28]. In our model the site is phosphorylated upon EGF-stimulation but returns to the levels of (or slightly-below) DMSO control when the drug is applied (Fig 4B), a negative fold change of approximately 1.4 relative to EGF-stimulation sample. This reversal suggests that SHP099 not only blocks its intended target in the signaling pathway (RAS/MAPK), but also indirectly modulates signaling at FOXO3:S7, which is associated with the PI3K-AKT pathway. These are traditionally considered distinct signaling routes, but our results indicate that the drug has more wide-reaching effects. Importantly, this phosphosite is not downregulated by other drugs, and in the remaining samples its levels are comparable to those seen with EGF stimulation (S16 Fig), underscoring the specificity of this effect and the model’s ability to predict novel drug responses that are absent from the training data. To explore the underlying mechanism, we made use of the trained network structure and node connections from the OmniPath prior knowledge network. We pruned the signaling network by excluding edges with weights below ±0.1 and ran a sensitivity analysis confirming that model performance was hardly affected by this (S17 Fig). Using this threshold, we calculated the shortest signaling distances from the drug target to the node of interest (FOXO3) to map potential signal propagation paths. While the drug target, PTPN11, is located within the RAS-ERK pathway, and FOXO3 in PI3K/AKT, the network analysis suggests that FOXO3’s signaling node may be connected downstream of PTPN11 via the p38 MAPK pathway, providing a plausible route for signal crosstalk, and MAPK14:T180 from this path indeed showed a similar regulation pattern (Fig 4A). The observed modulation of FOXO3:S7 by SHP099 exposes the complex regulatory networks governing FOXO3 activity.

### Kinase-substrate relation inference

We next assessed whether the model could be used to infer kinase–substrate relations. Using the pretrained network, we performed in silico inhibition of 31 human kinases that were also present in the training data. To ensure a fair comparison, we restricted the analysis to phosphosites that were both represented in our phosphoproteomic dataset and present in the OmniPath kinase-substrate database [20], which was not used in the modeling. For each kinase perturbation, phosphosites were classified as validated (both downregulated in the in-silico perturbation and present in the reference database), predicted only (predicted by the model but not listed in the reference and therefore treated as candidate interactions requiring experimental validation), or database only (listed in OmniPath but not predicted by the model).

**Fig 5:**
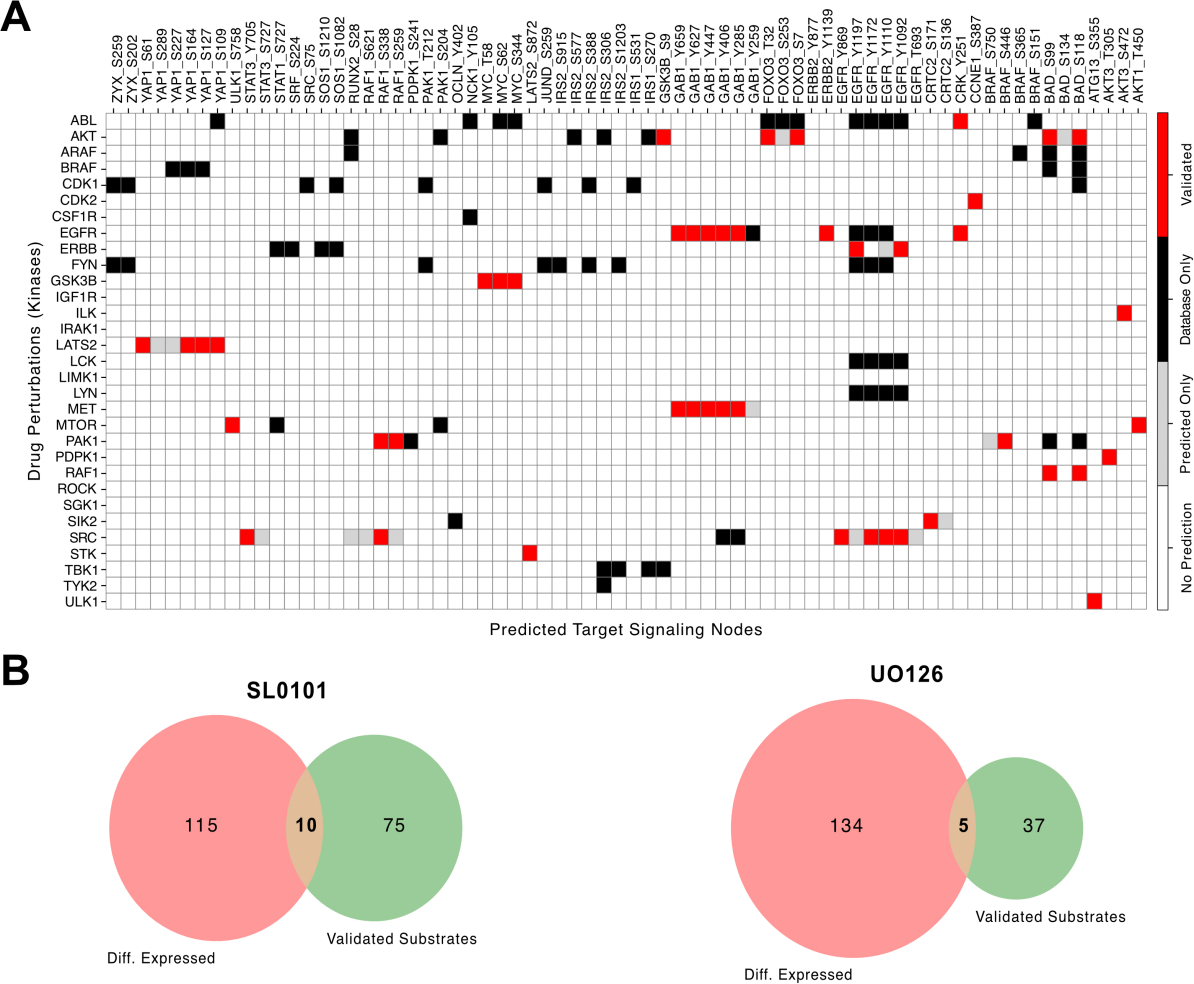
Kinase–substrate inference. **A.** Heatmap showing the overlap between predicted kinase–substrate pairs and validated pairs from OmniPath. Red indicates validated overlaps, while grey marks pairs predicted by the model but not present in OmniPath. Predictions are defined as phosphosites differentially expressed in in silico perturbations that are directly connected to the drug target in the OmniPath network. **B.** Overlap between differentially expressed phosphosites in the experimental data and validated kinase–substrate pairs for two drugs with available measurements.

The majority of the predictions were validated phosphosites that matched known kinase-substrate pairs, indicating a low false-positive rate under this conservative definition, while the model-only predictions represent putative novel targets (Fig 5A). There were also a comparable number of interactions that were not predicted in this study, reflecting false negatives that could be expected given the limited phosphosite coverage, cell-line specificity, and the requirement for an observable downregulation under the simulated perturbation. Important for this analysis was, the self-imposed constraint to only inspect direct connections, which prevents spurious assignments, ensuring that signaling nodes themselves were not misclassified as substrates but this necessarily trades sensitivity for specificity.

To highlight the advantage of a model driven analysis approach, we implemented a naïve inference strategy using only the experimental data. Differential expression analysis of two inhibitors versus EGF stimulation identified perturbed phosphosites, which were then compared against the kinase–substrate reference. In this case, overlap with validated pairs was severely limited (Fig 5B), highlighting the value of our integrative approach. For the remaining two inhibitors the overlap was essentially absent with only 1 database-listed kinase-substrate pair in total (S18 Fig). This is consistent with the fact that the targets of these drugs lack directly phosphorylated substrates in the OmniPath reference database and instead act through other types of interactions. For example, the target proteins of LY294002 (PI3K family) phosphorylate small molecules that activate downstream proteins, such as the AKT kinase [29]. Nevertheless, the model is still able to capture drug effects because the OmniPath network encodes diverse interaction types at the protein level, which are subsequently translated into phosphosite-level predictions by the phosphosite mapping layer.

## Discussion

We have extended the LEMBAS framework to model time resolved phosphoproteomic trajectories. This involved accounting for the full sequence of recurrent states by adding two interpretable modules: a phosphosite mapping that projects node activities to measured sites, and a monotonic time mapping that aligns continuous experimental times to discrete RNN steps. On synthetic benchmark tests the addition of each module improved the framework’s ability to explain variance in the data. Training the model using a time-series dataset of EGF stimulated mammary epithelial cells (MCF10A) from literature, it recovered held out time points including full interpolation using only data from 0 and 12 minutes and produced zero shot predictions of drug responses that outperformed a simple baseline model for three of four inhibitors. Importantly, achieving zero-shot generalization in this context is practically impossible for traditional statistical models lacking prior biological knowledge, as the limited set of perturbations available in the current context provides insufficient signal for purely data-driven models to infer the behavior of unseen inhibitors. The model furthermore identified plausible effects of drugs on both canonical and non-canonical downstream signaling states and produced kinase substrate candidate lists that substantially overlapped with curated OmniPath pairs.

The observation that the model can reliably interpolate intermediate time points, suggests a practical experimental strategy where data at these time points can be substituted with model predictions. For this we found it helpful to use single dimensional control measurements with higher time resolution, to act as anchor for the high-dimensional samples. This design allows a mapping between RNN steps and real time, and the model can then make use of more sparsely placed observations, to infer the remaining dynamics, ideally reducing the cost while retaining temporal resolution. However, this may be contingent on the quality of the high-resolution data, for example in our interpolation experiment we found that the 2-minute point was particularly difficult to predict accurately. This type of challenges are common for early time points, which exhibit comparably small net changes, higher technical noise, and greater replicate variability, and disturbing effects from sampling has a larger relative effect close to the stimulation timepoint [30]. In practice this may suggest that an increased number of replicates or denser sampling around early times when feasible or designing controls that better capture rapid transient kinetics.

To evaluate the model performance, a key challenge was to disentangle the baseline state from perturbation induced responses. For example, to isolate drug specific effects, we benchmarked on differential responses by subtracting appropriate baselines (EGF control at 12 minutes for zero-shot drug prediction). This focused the evaluation on inhibitor induced changes rather than shared stimulation signals, which would inflate the validation metrics. However, subtraction changes covariance and variance structure and can bias correlations [31]. As we observed, when the held-out drug’s raw signal was less correlated with the control, subtraction removes less shared variance and a naïve mean baseline may retain more of the remaining signal, superficially inflating its post-subtraction correlation. Thus, benchmarking by subtraction emphasizes differential effects but can change relative rankings in ways that reflect baseline covariance rather than model quality. We mitigate this by always comparing to a naïve estimation and using bootstrap tests for significance. Nevertheless, careful interpretation is required, and alternative baselines or complementary metrics should be considered in future studies.

A limitation of the current implementation is the ability to generalize to phosphosites not present in the training data. In the present setup the model can only predict phosphosites that explicitly appear in the training data, which prevents straightforward integration of public datasets where identifier conventions and detected site lists differ. A practical path forward may be to replace the current per-site learned embedding vectors with sequence informed embeddings that incorporate the peptide or protein sequence and the residue position of the phosphorylation [32,33]. By projecting each measured peptide into a learned sequence space, the model could potentially map novel or differently annotated phosphopeptides onto the same functional manifold, allowing prediction for sites unseen during training and removing the requirement for identical site lists across datasets.

A related limitation arises from the fact that phosphoproteomic measurements can, in some cases, be associated with multiple potential protein sites or functional contexts. [34]. In the dataset used in this works, such ambiguities had already carefully been addressed through prior-knowledge guided phosphosite remapping, providing a well-curated site-level representation for model training [23]. Building on this foundation, future extensions could benefit from introducing additional flexibility at the modeling stage, allowing phosphosite representations to softly integrate information from multiple signaling nodes when warranted by the data. This could be achieved by enabling site embeddings to attend to more than one node activity or by learning soft site-node assignments, allowing the model to reconcile residual uncertainty and context-dependent signaling while remaining consistent with curated phosphosite annotations.

Although we no longer restrict evaluation to a single steady state, but rather return all RNN states, the model retains a steady-state constraint to ensure convergence. This constraint reduces the ability to represent sustained oscillatory or non-contracting dynamics. Moreover, our zero-shot benchmarking depends on the available perturbations and network coverage. Predictions are expected to be more reliable for targets placed in well-connected regions of the prior network and for perturbations that resemble those seen in the training set.

Here we were able to accurately predict phosphorylation patterns over time and in response to drugs using a carefully curated and uniquely valuable phosphoproteomic time-series dataset. While this dataset enables systematic benchmarking of dynamic and drug-conditioned predictions, a major bottleneck for biologically informed learning more broadly remains the limited availability of similarly structured, tabulated, and machine-ready phosphoproteomic time series that span diverse perturbations and time scales [35–37]. The dataset used in this study is notable for providing both dense, early temporal sampling and a variety of inhibitor perturbations, a combination that remains rare in the field [23]. Our analysis using synthetic data suggests that predictive performance scales significantly with the variety of drug perturbations and by increasing the number of unique drugs in the training set leads to improved performance in the zero-shot setting. To build models that generalize across contexts the field needs coordinated datasets with dense time sampling, multiple perturbations and doses, and measurements across diverse cell lines and conditions, all provided in standardized tables with clear site identifiers and metadata. Generating such resources will accelerate the development of transferable models and more robust inference of drug responses. We therefore encourage efforts to produce and share high quality, standardized phosphoproteomic time series with broad perturbation coverage.

## Materials and methods

### LEMBAS framework

The core model follows Nilsson et al. [19], which frames signaling as an interpretable recurrent network constrained by a curated prior knowledge network (PKN). The PKN is derived from OmniPath [20], each node represents protein and directed interactions are encoded in an adjacency matrix A. At each discrete step the state vector h is updated and outputs are obtained by a trainable projection from node states. All of the constraints from the original work are retained, including penalties for learning the wrong sign of the weight relative to the known mode of action (Activation/Inhibition) and a spectral radius regularizer to promote convergent dynamics.

Model training was performed using a GPU-optimized implementation of LEMBAS. Hyperparameter choices were guided by a comprehensive analysis reported previously [24]. Based on these recommendations, L2 regularization was set to 10^−5^, the spectral radius constraint was enforced using five power iterations per update, and the learning rate was set to 2 × 10^−3^. Models were trained for 5000 epochs, which was sufficient to ensure smooth convergence of the loss without evidence of instability or overfitting across all experiments. The model hyperparameters are summarized in S1 Table.

Two modules were added to map signal node level dynamics to measured phosphosites and to align discrete RNN steps with experimental times. These modules were designed to be differentiable, so they train end to end with the recurrent core, and to preserve interpretability by enforcing valid site to node associations.

#### Drug to target projection.

Drug perturbations are formulated as a sample by drug matrix x. The projected inputs are scaled by trainable drug–target weights. When a drug is applied, a large negative input (−5) is supplied; this pushes the targeted node activity toward zero in a smooth, differentiable manner, as the model uses a leaky linear activation for negative inputs.

#### Phosphosite mapping layer.

Node activities h (samples × recurrent steps × nodes) are first projected to site space via a site to node matrix P that maps each signaling node to its corresponding phosphosite (1). Each raw phosphosite input i is used to scale a trainable embedding vector $E_{i}$ (2). These are used as input for a multilayer perceptron MLP (shared between all phosphosites) that collapses the embedding to a scalar intensity for each site (3).


$$h_{site}=h\cdot P\;(\text{1}),\;S=h_{site}\cdot E\;(\text{2}),\;{\hat{Y}}_{site}=MLP\left(S\right)$$ (3)

All embeddings and MLP parameters are learned with L2-norm regularization [38].

#### Time point mapping layer.

Observed experimental times do not necessarily align with the discrete updates of the RNN states. We therefore learn a monotonic differentiable mapping from RNN steps to experimental time points via one learnable anchor per observed time. To ensure a monotone relation between RNN steps and timepoints, anchors are parameterized from raw offsets $\delta_{raw}$ with an upper bound parameter $u_{raw}$, to constrain the anchors to the predefined total number of RNN steps (L). The upper bound is computed as


$$u=L\cdot sigmoid\left(u_{raw}\right)$$ (4)

Anchors are formed by transforming $\delta_{raw}$ to strictly increasing positive values using softplus and cumulative sum, normalizing to [0,1], applying an exponential reweighting controlled by trainable α, and scaling by u:


$$anchors=u\cdot \frac{e^{\alpha }\cdot cumsum{\left(softplus\left(\delta_{raw}\right)\right)}_{norm}+\text{1}}{e^{\alpha }+\text{1}}$$ (5)

These mapped indices are continuous. For each mapped index m we obtain predictions by soft indexing between the two nearest integer RNN steps:


$$\hat{y}=\left(\text{1}-\Delta_{m}\right)\cdot h_{\left\lfloor m\right\rfloor }+\Delta_{m}\cdot h_{\left\lfloor m\right\rfloor }\;(\text{6}),\;\Delta_{m}=m-\left\lfloor m\right\rfloor$$ (7)

This interpolation preserves end to end differentiability through Δ.

### Synthetic data generation

Synthetic datasets were produced from a pretrained LEMBAS instance on a medium scale network (409 nodes) to allow controlled evaluation of model performance. Each synthetic drug is configured to inhibits a single node with a large negative constant (-5). All samples are modeled to received constant EGF-stimulation. Node to phosphosite relations were simulated by sampling per node site counts from an exponential distribution, assigning per site scaling factors, rescaling, and log2 transforming the node state. RNN trajectories were simulated for 150 steps and subsampled to emulate experimental sampling at t = 0,1,2,3,5,8,10,15,20,50.

### Optimization and loss profile

Models were trained with Adam with warm restarts and a cosine annealing learning rate schedule. The primary training objective was mean squared error (MSE) between predicted and observed site intensities. We applied smooth gradient clipping, L2 regularization on parameters, and additive Gaussian gradient noise whose amplitude decays with the learning rate. Mini batches contain all time points for a single sample to preserve temporal coherence. Missing site measurements and invalid site node pairs were masked and excluded from the MSE loss.

### Baselines and performance metrics

Performance is calculated as Pearson correlation between predicted and observed difference trajectories. For time series evaluations we subtract each trajectory’s timepoint zero to baseline-normalize dynamics and remove baseline offsets. For zero-shot drug prediction benchmarking we subtract the EGF control data and fit at the 12-minute time point from data and model outputs respectively prior to computing correlations. This focuses evaluation on drug induced differential effects relative to the stimulated control and removes shared activation that would otherwise dominate correlations. For zero shot drug baselining we compare against a naïve estimator that predicts the held-out drug as the mean of the remaining drugs. Statistical significance is assessed by bootstraping.

### EGF-stimulation dataset and preprocessing

An EGF-stimulation dataset was retrieved from literature [23,39]. From this study we used two subsets: a time series spanning 0–12 minutes after 1 ng/ml EGF-stimulation in MCF10A cells and phosphoproteome measurements at 12 minutes under four inhibitory drug conditions with and without EGF plus a DMSO control. All samples were available in two biological replicates. We used preprocessed data directly from the original publication [23]. One hot matrix for signaling node to phosphosite mapping and for sample to drug mapping were constructed from metadata and curated drug target annotations.

### OmniPath filtering

To reduce model complexity and focus inference on EGF-relevant signaling, the OmniPath network was pruned using networkX. We retained all nodes reachable from the EGF receptor node by any directed path, ensuring the subnetwork captured only signaling downstream of the stimulation input. This reduced the network from 2603 nodes to 2029, and the resulting subnetwork served as the adjacency matrix A during training.

## Supporting information

S1 Table Selected model hyperparameters. (XLSX)

S1 Fig Unit tests for phosphosite mapping. A. Test data illustrating four distinct relationships between signaling nodes and phosphosites: cosine, polynomial, sigmoid, and square root. B. Comparison of fitted values versus actual data points, along with correlation metrics, across various phosphosite layer configurations, including different embedding sizes and MLP architectures.
(TIF)

S2 Fig Example node-to-site relationships. (TIF)

S3 Fig Unit tests for time mapping. A. Test data showing four relationships between time index and phosphosite values (cosine, polynomial, sigmoid, square root) under three sampling schemes (exponential, random, uniform). B. Time index training across epochs; dashed lines indicate the true time indices. C. Assessment of whether combining sample types resolves issues with the cosine relationship under random or exponential sampling; training trajectories for different ratios of polynomial and cosine samples. D. Limitations with flat-line inputs; training trajectories for time index mapping using flat input signals.
(TIF)

S4 Fig Impact of normalization and scaling parameters on the relationship between anchor and final mapped values. (TIF)

S5 Fig Schematic overview of synthetic data generation pipeline. Synthetic datasets were generated using a pretrained LEMBAS model on a medium-scale (KEGG) network of 409 nodes. Each synthetic drug was configured to inhibit a single node with a strong negative effect (-5), while all samples received constant EGF stimulation. Node-to-phosphosite relationships were simulated by sampling site counts per node from an exponential distribution, assigning per-site scaling factors, rescaling, and log₂-transforming node states. RNN trajectories were then simulated for 150 steps and subsampled to mimic experimental measurements at t = 0, 1, 2, 3, 5, 8, 10, 15, 20, and 50 minutes.
(TIF)

S6 Fig Temporal down-sampling analysis of interpolation performance on synthetic data. Pearson correlation as a function of the percentage of uniformly spaced timepoints provided during training, evaluated on the first 30 RNN steps where primary signaling dynamics occur. Annotated with the amount of points available during training.
(TIF)

S7 Fig Scaling of zero-shot predictive performance. Mean Pearson correlation (r) for held-out drug predictions as a function of the number of unique drugs included in the training set. Each data point represents the average performance across a leave-one-drug-out cross-validation procedure.
(TIF)

S8 Fig Training loss curve for the model used to infer intermediate timepoints shown in Fig 2. (TIF)

S9 Fig Standard deviation of phosphosites across conditions based on network reachability. The distributions of the phosphosites standard deviation across conditions categorized by their connection to the EGF receptor in the prior knowledge network.
(TIF)

S10 Fig Pearson correlations for each individual timepoint across the different interpolation tasks presented in Fig 2. (TIF)

S11 Fig Differential signal variance across timepoints. The distributions of the experimental absolute differential phosphorylation fold-change relative to t = 0 for all measured phosphosites at 2 minutes and 8 minutes following EGF stimulation.
(TIF)

S12 Fig Zero-shot prediction of drug-specific phosphoproteomics responses. Model predictions versus experimental data (uncorrected).
(TIF)

S13 Fig Effect of lack of network pruning on zero-shot predictions. Model predictions versus experimental data for the unpruned network.
(TIF)

S14 Fig Stability of zero-shot predictions across model initializations. Distribution of the coefficient of variation (CV; standard deviation divided by mean) of predicted phosphosite intensities across ten independently trained models with different random initializations. CVs were computed for each phosphosite–sample combination using raw model predictions prior to baseline subtraction.
(TIF)

S15 Fig Zero-shot average prediction of drug-specific phosphoproteomics responses. A. Average model predictions versus experimental data. B. Distributions of phosphosites model predictions per model run (different seed) per drug.
(TIF)

S16 Fig Phosphorylation levels of FOXO3:S7 across two replicates. Full time series shown for EGF-stimulation; static data at 12 min for DMSO, SHP099 inhibition and other inhibitor samples. Line indicates means; ribbon shows standard deviations; dashed lines indicate DMSO-baseline and other inhibitor samples mean.
(TIF)

S17 Fig Sensitivity analysis for determining the threshold to zero out weights while preserving model performance. (TIF)

S18 Fig Overlap between differentially expressed phosphosites in the experimental data and validated kinase–substrate pairs for two drugs. (TIF)
